# The effect of vitamin D supplementation on antibiotic use: a meta-analysis based on randomized controlled trials

**DOI:** 10.3389/fnut.2024.1502835

**Published:** 2024-11-12

**Authors:** Mian Wang, Yixuan Wu, Zhiyi Xiang, Yueming Zhang, Tingyi Huang, Bangsheng Chen

**Affiliations:** ^1^Infection Department, Ningbo Yinzhou No. 2 Hospital, Ningbo, China; ^2^The Second Clinical Medical College, Zhejiang Chinese Medical University, Hangzhou, Zhejiang, China; ^3^The First Clinical Medical College, Zhejiang Chinese Medical University, Hangzhou, Zhejiang, China; ^4^Intensive Care Unit, Hospital of Zhejiang People's Armed Police, Hangzhou, Zhejiang, China; ^5^Emergency Medical Center, Ningbo Yinzhou No. 2 Hospital, Ningbo, China

**Keywords:** vitamin D supplementation, antibiotic use, infection, respiratory tract infections, vitamin D deficiency, meta-analysis

## Abstract

**Objective:**

This meta-analysis explores the impact of vitamin D supplementation on antibiotic utilization.

**Methods:**

We systematically searched for relevant randomized controlled trials (RCTs) in PubMed, Web of Science, EMBASE, and Science Direct from inception to April 2024. These trials compared antibiotic use rates between groups receiving vitamin D supplements and placebo.

**Results:**

We included seven RCTs involving 35,160 participants. There was no significant difference in antibiotic use between the two groups in the general population (Odds Ratio [OR] = 0.98, *p* = 0.232), including elderly participants (OR = 0.98, *p* = 0.295). However, antibiotic use was lower in the intervention group compared to the placebo group among participants under 70 years of age (OR = 0.95, *p* = 0.015), those with relative vitamin D deficiency [25(OH)D < 75 nmol/L, OR = 0.95, *p* = 0.024; 25(OH)D < 50 nmol/L, OR = 0.96, *p* = 0.026], and those with respiratory tract infections (RTIs) (OR = 0.51, 95% CI: 0.24–1.08, *p* = 0.080), although these differences were not statistically significant for RTIs.

**Conclusion:**

Vitamin D supplementation does not affect antibiotic use in the general population. However, it does reduce antibiotic utilization in individuals with RTIs, relative vitamin D deficiency, or aged below 70 years.

**Systematic review registration:**

This meta-analysis adheres to the Preferred Reporting Items for Systematic Reviews and Meta-Analyses (PRISMA) guidelines, and is registered with the International Prospective Register of Systematic Reviews (PROSPERO), registration number CRD42024543246.

## Introduction

A spatial modeling study published in 2021 reported that global antibiotic consumption increased by 46% from 2000 to 2018, peaking at 40.1 defined daily doses (DDDs) in 2018 (1). Antibiotics, pivotal in reducing the morbidity and mortality associated with many infectious diseases, are considered life-saving drugs. However, their widespread availability and perceived cost-effectiveness have led to increased irrational and misuse (2). This is compounded by a lack of adequate awareness among both the public and medical professionals. Such overuse has accelerated the development of drug-resistant bacteria, posing a significant threat to global health due to the ensuing antibiotic resistance (3).

In response to the critical issue of antibiotic resistance, no effective alternatives have been developed, which necessitates the continuous development of new antibiotics (4). Recent studies suggest that combining antibiotics with non-antibiotic compounds could improve treatment outcomes against multi-resistant bacteria by possibly aiding in antibacterial action or repairing metabolic defects (5). For instance, it has been demonstrated that the addition of substrates like glucose or alanine can enhance the tricarboxylic acid cycle, thereby increasing bacterial uptake of antibiotics and improving their efficacy (6). Additionally, existing studies have suggested that vitamin D deficiency also played an important impact on extra-skeletal diseases, especially on respiratory tract infections (RTIs) such as bacterial pneumonia and acute respiratory infections (ARIs) (7). Notably, vitamin D has been recognized for its substantial immunomodulatory effects, such as activating immune cell chemotaxis, enhancing phagocytic capabilities of macrophages (8), and inducing the production of antimicrobial peptides (9). These properties suggest that vitamin D could serve as a supportive antimicrobial agent. A prior meta-analysis involving 25 randomized controlled trials (RCTs) indicated that vitamin D supplementation could lower the incidence of ARIs (10). Moreover, a prospective observational study in Sweden showed that vitamin D supplementation was associated with a reduction in antibiotic usage days (11). There was, in addition, a cohort study shown that low serum vitamin D levels were an independent predictor of adverse outcomes of COVID-19 and might result in higher levels of inflammation and more serious tissue damage in patients with severe or non-severe cases (12). Despite these findings, there were no meta-analyses that explore the effect of vitamin D supplementation on antibiotic use. In light of these considerations, this study aims to review published RCTs to perform a meta-analysis assessing the relationship between vitamin D supplementation and antibiotic usage frequency in adults.

## Methods

### Search strategy

This meta-analysis adheres to the Preferred Reporting Items for Systematic Reviews and Meta-Analyses (PRISMA) guidelines (13), and is registered with the International Prospective Register of Systematic Reviews (PROSPERO), registration number CRD42024543246. We conducted a systematic search for RCTs examining the effects of vitamin D supplementation on antibiotic use from inception to April 2024. Searches were performed using PubMed, Web of Science, EMBASE, and Science Direct, with keywords including (vitamin D) AND (antibiosis OR antibiotic OR antibiotics OR anti-infection OR infection). A secondary search was conducted through the references of all identified studies to ensure the comprehensiveness of our search.

### Selection and exclusion criteria

The inclusion and exclusion of studies were guided by the PICOS (participants, intervention, comparison, outcomes, and study design) framework (14). The inclusion criteria were as follows: (1) participants: adults aged 16 years or older, or those at high risk of antibiotic use due to certain diseases (excluding tuberculosis); (2) intervention: oral administration of vitamin D in the intervention group; (3) comparison: placebo given to the control group; (4) outcomes: measures related to antibiotic use; (5) study Design: only RCTs were considered.

Exclusion criteria included: (1) studies where relevant data could not be extracted or were unsuitable for statistical analysis; (2) studies where the full text was unavailable; (3) studies not published in English; (4) studies with outdated or superseded publications, where articles with the most recent and comprehensive data were given preference.

### Data extraction and quality assessment

Data extraction was performed independently by two investigators using a predefined form. This form captured essential information including the authors, year of publication, country, clinical trial number, participant characteristics (age, number, recruitment year, physical condition, and vitamin D supplementation regimen), and antibiotic-related outcomes.

Risk of bias was assessed according to the guidelines provided by the Cochrane Collaboration Network (15). The assessment covered several domains: (1) random sequence generation (to address selection bias), (2) allocation concealment (to address selection bias), (3) blinding of participants and personnel (to mitigate performance bias), (4) blinding of outcome assessment (to mitigate detection bias), (5) completeness of outcome data (to address attrition bias), (6) selective reporting (to address reporting bias), and (7) other potential biases. Each domain was rated as ‘high risk,’ ‘low risk,’ or ‘unclear risk’. Disagreements between investigators were resolved through discussion to reach a consensus.

### Statistical analysis

Statistical analyses were conducted using Stata Software version 12.0 (Stata Corporation LLC, College Station, United States). The impact of vitamin D supplementation on antibiotic use was evaluated using odds ratios (ORs) and 95% confidence intervals (CIs). Heterogeneity among the studies was assessed with a chi-square test and quantified using the *I*^2^ statistic. *I*^2^ values over 50% were considered indicative of significant heterogeneity, values between 25 and 50% indicated moderate heterogeneity, and values below 25% indicated low heterogeneity (16).

Due to potential variations among study participants and differences in study protocols, analyses were performed using a random-effects model to enhance the reliability of the results. Subgroup analyses were conducted to explore the sources of heterogeneity further. Publication bias was assessed using Begg’s test, and sensitivity analyses were performed to verify the stability of the findings. All statistical tests were two-sided, with a significance threshold set at *p* < 0.05.

## Results

### Study selection

**Figure 1:**
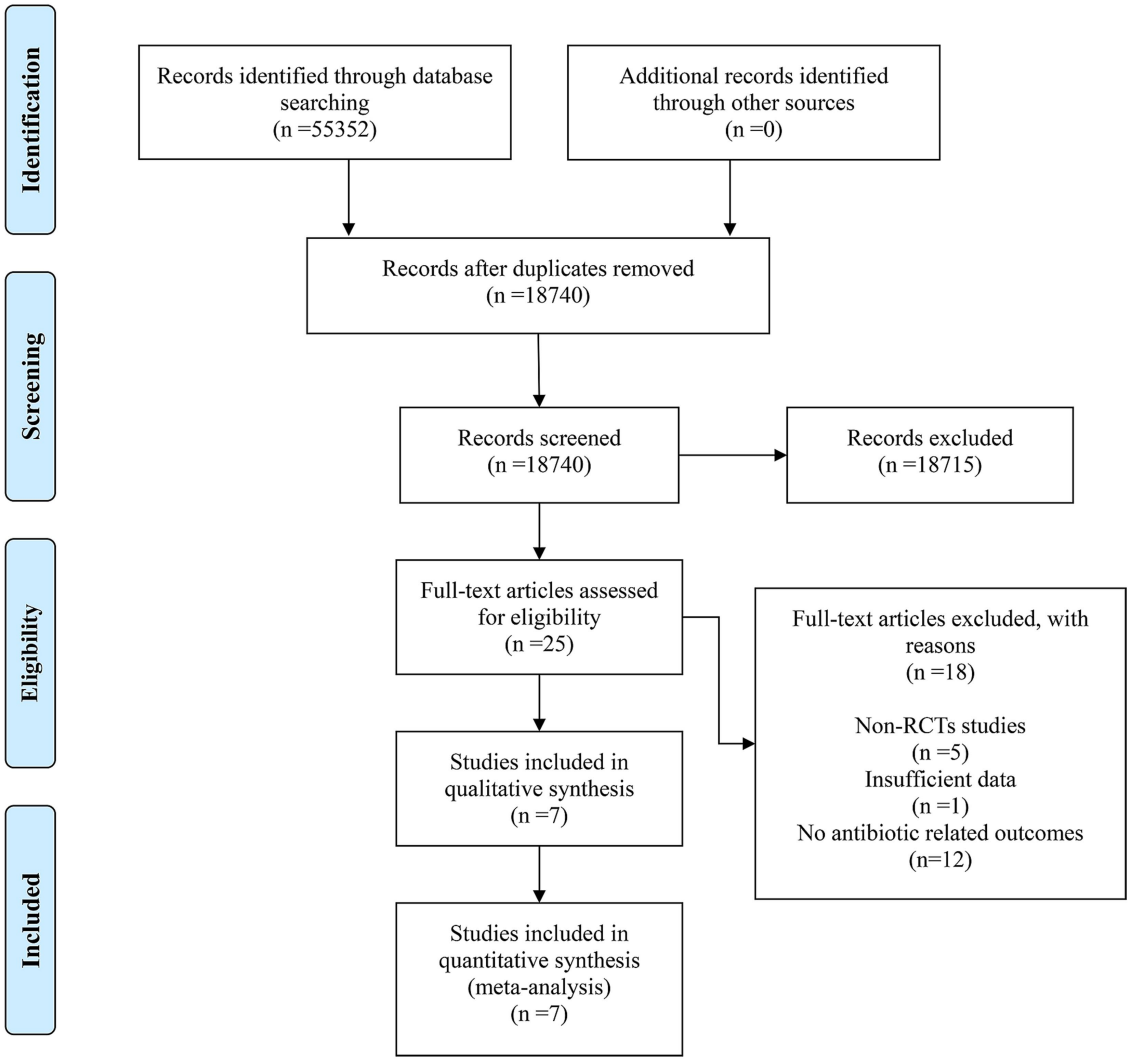
Flow chart of study selection.

From four electronic databases, a search identified 55,352 records under the specified research strategy. No additional records were identified through other sources. After removing duplicates, 18,740 records remained. Screening of titles and abstracts led to the exclusion of 18,715 records due to low relevance, leaving 25 full-text articles for detailed evaluation. Out of these, 18 articles were excluded for the following reasons: 5 were non-RCTs, 1 had insufficient data, and 12 did not report antibiotic-related outcomes. Ultimately, 7 studies met the inclusion criteria and were included in the meta-analysis. The detailed retrieval process is illustrated in Figure 1.

### Characteristics and quality assessment of included studies

The 7 RCTs (17–23), spanning from 2007 to 2022 and cited as references, investigated the relationship between vitamin D supplementation and antibiotic use, involving 35,160 participants from five countries (Sweden, United Kingdom, Australia, Netherlands, New Zealand). The intervention groups in these studies received oral vitamin D supplementation, while control groups were administered a placebo. Six (17, 18, 20–23) of the studies involved oral cholecalciferol and one (19) involved oral D-Peals capsules produced in Denmark. Duration of intervention varied: three studies (17, 20, 23) had durations exceeding 1 year, and the remaining four (18, 19, 21, 22) were conducted for 1 year or less.

Participants’ physical conditions varied across studies: one (18) involved participants with antibody deficiencies or frequent RTIs; one (19) included individuals with 25(OH)D levels below 75 nmol/L; one (21) focused on patients with a history of chronic obstructive pulmonary disease (COPD) exacerbation within the last 12 months and 25(OH)D levels below 50 nmol/L; one (17) targeted patients with low trauma and osteoporotic fractures; and three (20, 22, 23) included elderly individuals from the general community. Further characteristics and details of the included studies are presented in Table 1 and Supplementary Table S1.

**Table 1:** Characteristics of all the studies included in the meta-analysis.

Author	Year	Country	No. of participants		Participant characteristics		Dosage and duration of Vitamin D
			Vitamin D	Placebo	Age (year)	Physical condition	
			Male/Female				
Bergman, Peter	2012	Sweden	18/52	20/50	18–75	Antibody deficiency or frequent RTIs	4,000 IU daily for 1 year
Jolliffe, David A.	2022	UK	498/1052	1040/2060	≥16	25(OH)D < 75 nmol/L	800 IU daily for 6 months
			506/1044				3,200 IU daily for 6 months
Pham, Hai	2022	Australia	5336/4426	5327/4408	60–84	/	60,000 IU monthly for 5 years
Rafiq, R.	2022	Netherlands	46/28	55/26	≥40	A COPD exacerbation in the last 12 months before screening, 25(OH)D < 50 nmol/L	16,800 IU weekly for 1 year
Tran, Bich	2014	Australia	113/97	110/95	60–84	/	30,000 IU monthly for ≤12 months
			109/96				60,000 IU monthly for ≤12 months
Wu, Zhenqiang	2021	New Zealand	1512/1046	1457/1093	50–84	/	100,000 IU monthly for a median of 3.3 years
Avenell, A.	2007	UK	1737	1703	≥ 70	Low trauma, osteoporotic fracture	800 IU daily for 18 months

RTIs, respiratory tract infections; No, number; COPD, chronic obstructive pulmonary disease.

The risk of bias was assessed for each study using the Cochrane Collaboration’s tool, as depicted in Supplementary Figures S1, S2. One study (19) was deemed to have a high risk of selection and performance bias due to inadequate concealment of treatment allocation and lack of stratified randomization. Another study (21) was identified as having a high risk of bias due to not achieving the designed sample size. Three studies (18, 22, 23) presented challenges in determining other biases. Overall, the studies were considered to be of high quality (Table 2).

**Table 2:** Subgroup analysis of the effect of vitamin D supplementation on antibiotic use.

Subgroup	No. of studies	OR (95% CI)	*P*	*I* ^2^
Age				
Older adults	4	0.98 [0.96, 1.01]	0.295	31.8%
≥70	3	0.99 [0.96, 1.03]	0.731	23.7%
<70	2	0.95 [0.91, 0.99]	0.015	0.0%
25(OH)D concentration				
<75 nmol/L	4	0.95 [0.92, 0.99]	0.024	0.0%
<50 nmol/L	3	0.96 [0.92, 0.99]	0.026	0.0%
Doses of vitamin D				
> 2000 IU/day	4	0.95 [0.66, 1.36]	0.765	49.4%
≤2000 IU/day	4	0.88 [0.75, 1.03]	0.111	39.0%
Supplementation time of vitamin D				
>1 years	3	0.99 [0.97, 1.00]	0.109	0.0%
≤1 year	4	0.77 [0.52, 1.15]	0.205	38.7%
Infection type				
RTIs	2	0.51 [0.24, 1.08]	0.080	22.4%

RTIs, respiratory tract infections; OR, odds ratio; CI, confidence interval; No, number.

### Analysis of the primary result

**Figure 2:**
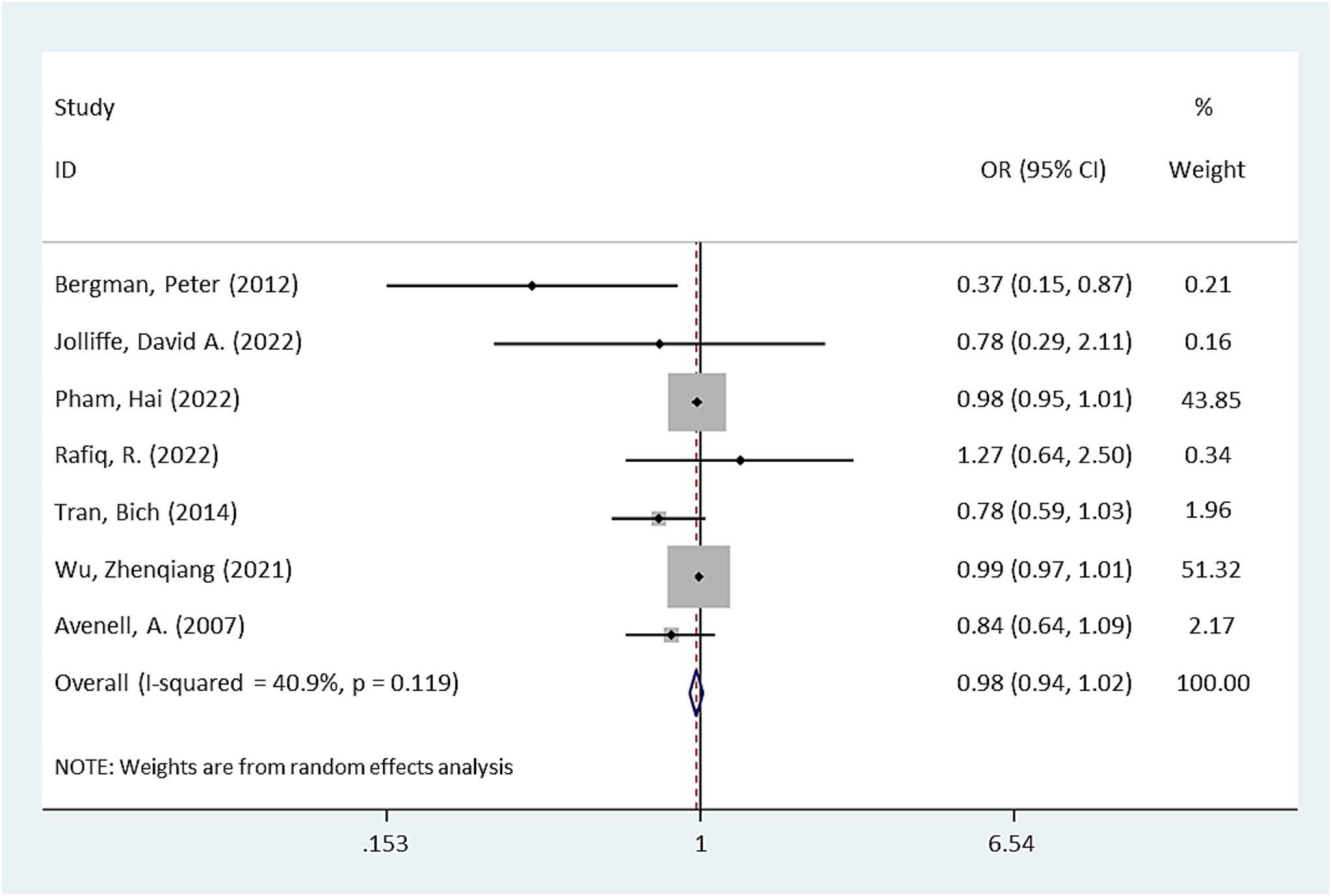
Forest plot of the effect of vitamin D supplementation on antibiotic use (*p* = 0.232).

Pooling the results from seven RCTs (17–23), no significant difference in antibiotic use was observed between the intervention group receiving vitamin D supplementation and the placebo group (OR = 0.98, 95% CI: 0.94–1.02, *p* = 0.232, Figure 2). However, there was moderate heterogeneity among the studies (I^2^ = 40.9%).

### Subgroup analysis

Subgroup analyses were conducted based on participant age thresholds. For participants aged ≥70 years, no statistical difference in antibiotic use was observed between the intervention and control groups (OR = 0.99, *p* = 0.731). Conversely, among participants aged <70 years, the intervention group exhibited a reduced use of antibiotics compared to the control group (OR = 0.95, *p* = 0.015). Additional subgroup analysis among older adults similarly showed no significant differences in antibiotic use between the groups (OR = 0.98, *p* = 0.295).

Vitamin D concentration levels of less than 75 nmol/L or 50 nmol/L were considered relatively inadequate (24). Among participants with 25(OH)D levels <75 nmol/L, four RCTs (19–21, 23) indicated reduced antibiotic use in the intervention group compared to the control group (OR = 0.95, *p* = 0.024). For participants with 25(OH)D levels <50 nmol/L, results from three RCTs (17, 18, 22) demonstrated that the vitamin D-receiving group used fewer antibiotics than the placebo group (OR = 0.96, *p* = 0.026), with no significant heterogeneity (*I*^2^ = 0%).

Regarding vitamin D dosage, participants were categorized based on daily intake exceeding 2000 IU (high-dose supplementation group) or not (low-dose supplementation group). Neither the high-dose group (OR = 0.95, *p* = 0.765) nor the low-dose group (OR = 0.88, *p* = 0.111) showed significant differences in antibiotic use. Similarly, based on the duration of supplementation, no significant differences were found either for durations greater than 1 year (OR = 0.99, *p* = 0.109) or less than or equal to 1 year (OR = 0.77, *p* = 0.205).

For participants suffering from RTIs in two RCTs (18, 19) the intervention group exhibited a lower rate of antibiotic utilization compared to the control group, though the difference was not statistically significant (OR = 0.51, 95% CI: 0.24–1.08, *p* = 0.080).

### Publication bias and sensitivity analysis

Publication bias was assessed using Begg’s test, which indicated no significant bias in the results related to the effect of vitamin D on antibiotic use (*p* = 0.230, Supplementary Figure S3). Sensitivity analysis was conducted by sequentially excluding each study, confirming that the results remained stable (Supplementary Figure S4).

## Discussion

In this meta-analysis of seven RCTs (17–23), no significant association was observed between vitamin D supplementation and the risk of antibiotic use, encompassing elderly participants and various subgroup analyses concerning dosage and duration of vitamin D supplementation. However, among participants under 70 years of age, those with relative vitamin D deficiency, or those suffering from RTIs, vitamin D supplementation appears to reduce antibiotic usage.

Vitamin D, recognized as a multifunctional health-promoting molecule (25), is absorbed into the bloodstream and converted in the kidneys to its active form, 1,25(OH)2D3, via the catalytic action of 1α-hydroxylase (26). It primarily exerts its effects through interaction with the vitamin D receptor (VDR), facilitating the receptor complex’s migration to the nucleus and modulating the expression of numerous genes related to immune regulation and infection control (27). The immunomodulatory mechanisms of vitamin D include enhancing phagocytosis and chemotaxis of innate immune cells such as macrophages and monocytes, thereby improving pathogen clearance (28); inducing dendritic cell tolerance through the expression of CYP27B1, which enhances localized concentrations of active vitamin D at infection sites (29); and integrating with pattern recognition receptors (PRRs) to detect microbial signals and activate downstream infection-fighting pathways (30). Additionally, vitamin D helps regulate inflammatory responses by inhibiting pro-inflammatory cytokines like interleukin-2 (IL-2) and promoting the production of anti-inflammatory cytokines such as IL-10 (31).

Beyond immune regulation, vitamin D also plays a significant role in anti-infection processes: it promotes the production of host defense peptides, including cathelicidin antimicrobial peptide (CAMP) and human β2-defensins (Defb2) (32), with vitamin D response elements directly influencing their gene expression (33). It mediates the synthesis of nitric oxide (NO), which enhances antimicrobial activity (34) and may reduce the viability of *Streptococcus pneumoniae* and the emergence of antibiotic resistance (35). Furthermore, vitamin D reduces the activity of mammalian target of rapamycin (mTOR), supports the recruitment of ATG16L1 by NOD2 to induce autophagy, and aids in the elimination of intracellular bacteria (36). The antioxidant properties of vitamin D help eliminate harmful reactive oxygen species (ROS), moderating inflammation and maintaining mitochondrial function, which is crucial in reducing TNF-*α*-induced lung epithelial inflammation and mitochondrial autophagy (37, 38). In conclusion, while vitamin D’s role in reducing antibiotic use was not uniformly observed across all study participants, its various immunomodulatory and antibacterial properties theoretically support the reduction of antibiotic usage, particularly in certain subpopulations.

Although the mechanisms discussed were not substantiated across all participants in our study, several factors could explain these findings. Firstly, certain disease states and environmental factors might suppress antimicrobial peptide levels, thus diminishing the anti-infective efficacy of vitamin D. Studies have demonstrated that diabetes can down-regulate the expression of antimicrobial peptides (39), and prolonged exposure to polluted air reduces these peptide levels in mice (40). Secondly, genetic variations in the VDR genes affect individual responsiveness to vitamin D. A meta-analysis has shown that genotypes with the TaqI polymorphism and the FF variant in the FokI gene are more responsive to vitamin D supplementation (41). Thirdly, in individuals who are not deficient in vitamin D, additional supplementation may lead to inefficient binding of the vitamin to its receptors, as excess vitamin D is converted to 1,24,25(OH)2D3, which has minimal affinity for VDR (42). This hypothesis is supported by subgroup analysis indicating that high-dose vitamin D supplementation does not significantly reduce the risk of antibiotic use. This is consistent with a single-center RCT finding that additional vitamin D supplementation did not decrease hospital-acquired infection rates among sepsis patients (43), suggesting that vitamin D supplementation may not universally contribute to reduced antibiotic use.

Moreover, while one study suggested that high doses of vitamin D could decrease inflammation levels and enhance anti-infection capabilities (44), our subgroup analysis found no significant benefits from either low or high doses of vitamin D supplementation in reducing antibiotic use. Possible explanations include: (1) active vitamin D maintains a dynamic equilibrium in the body, with excess being converted to an inactive form that cannot be effectively utilized (42); (2) variability in the effectiveness of different vitamin D supplementation regimens; and (3) the prevalence of adequate serum vitamin D levels among our study participants, which could obscure any potential benefits for those with insufficient vitamin D levels. Previous studies have demonstrated that vitamin D supplementation had a more phenomenal impact on participants with vitamin D deficiency (45).

Emerging evidence underscores the association between low serum vitamin D levels and increased infection risk (46–48). The Third National Health and Nutrition Examination Survey demonstrated an inverse relationship between serum vitamin D levels and recent upper respiratory tract infections in the American population (49). Furthermore, a meta-analysis confirmed that vitamin D deficiency heightens susceptibility to serious infections (50). Our subgroup analysis for participants with low serum vitamin D levels (25(OH)D < 50 nmol/L or <75 nmol/L) corroborates these findings, suggesting that vitamin D deficiency may compromise neutralizing antibody production and immune cell function (51). Therefore, vitamin D supplementation could potentially enhance immune responses and infection resistance.

Specifically, studies indicate that antibiotic usage is more prevalent among the elderly, women (52), and individuals in poorer health (53). Despite the theoretical benefits of vitamin D in boosting immunity among the elderly to combat infections, our findings did not support this hypothesis for participants aged ≥70 years. This discrepancy may be attributable to several factors: (1) age-related decline in organ function associated with calcium metabolism may lead to decreased expression of VDR, resulting in inefficient utilization of vitamin D (54); (2) the prevalence of chronic kidney disease in older adults impairs the kidneys’ ability to activate vitamin D (55); (3) parathyroid hormone, known to enhance the synthesis of 1α-hydroxylase (56)—which converts vitamin D to its active form—is often diminished in older women, as evidenced by higher rates of hypoparathyroidism in this group (57); (4) comorbid conditions such as diabetes can negatively affect antimicrobial peptide production (39). A meta-analysis involving 41,552 elderly patients revealed that vitamin D supplementation did not significantly reduce the incidence of ARIs or lower respiratory infections (58), further supporting our observations.

Conversely, vitamin D supplementation was found to be beneficial in participants under 70 years of age. Possible explanations include: (1) younger individuals often engage in higher levels of physical activity, which may enhance vitamin D metabolism in adipose tissue (59); (2) higher physiological requirements and lower dietary intake of vitamin D in younger populations may lead to more pronounced deficiencies (60, 61), which supplementation can effectively address.

As for the type of infection, many studies available now have confirmed that vitamin D can relieve the symptoms of infection or reduce the onset of RTIs. This was consistent with our findings. The reasons may be as follows: (1) vitamin D can promote the repair of epithelial cells and inhibit the apoptosis of epithelial cells, thereby improving lung function (62); (2) vitamin D can enhance mucosal immunity including respiratory mucosa (63); (3) existing study showed that daily supplementation of vitamin D could increase the antibacterial activity of airway surface fluids (37); (4) RTIs activated T and B lymphocytes and significantly up-regulated the expression of VDR (64, 65). Thus, vitamin D supplementation can conduce to the promotion of the ability to fight infection and reduce the risk of antibiotic use in people suffering from RTIs.

For all we know, this was the first meta-analysis to conduct a comprehensive and systematic exploration of the relationship between the antibiotic use and the supplementation of vitamin D. It could provide a reference value for the field of antibiotic use. Importantly, the meta-analysis was based on the RCTs with high quality. Nevertheless, there were some limitations in the present meta-analysis. Firstly, the number of studies was limited. Besides, on account of the limited data, we could not perform further subgroup analysis including the sex or the body mass index. Moreover, certain heterogeneity was produced due to the different physical conditions of the subjects and various programs of vitamin D supplementation.

## Conclusion

This meta-analysis revealed that vitamin D supplementation does not significantly impact antibiotic usage in the general population, including elderly individuals. The regimen of vitamin D supplementation also showed no effect on antibiotic use. However, vitamin D supplementation may be beneficial in reducing antibiotic use among individuals under 70 years of age, those with relative vitamin D deficiency, or those suffering from RTIs. To substantiate these findings, more multicenter RCTs on a larger scale are necessary.

## Data Availability

The raw data supporting the conclusions of this article will be made available by the authors, without undue reservation.

## Supplementary material

SUPPLEMENTARY FIGURE S1 Risk of bias graph for quality assessment of the included RCTs.

SUPPLEMENTARY FIGURE S2 Risk of bias summary for quality assessment of the included RCTs.

SUPPLEMENTARY FIGURE S3 Begg’s test for assessing publication bias of included RCTs (*p*=0.230).

SUPPLEMENTARY FIGURE S4 Sensitivity analysis for testing the stability of statistical results.
