# Supplementary material for: The effect of vitamin D supplementation on antibiotic use: a meta-analysis based on randomized controlled trials
Source: Front Nutr. 2024 Nov 12;11:1502835. doi: 10.3389/fnut.2024.1502835 (PMC11588496; doi:10.3389/fnut.2024.1502835)
Supplement: Supplementary file 1 [file Table_1.DOCX]

**Supplementary Table 1. Characteristics of all the studies included in the meta-analysis.**

| Author | Year | Clinical trial number | Recruitment year | Antibiotic-related outcomes |
| --- | --- | --- | --- | --- |
| Bergman, Peter | 2012 | NCT01131858 | 2010 | Participants with antibiotic use |
| Jolliffe, David A. | 2022 | NCT04579640 | 2020 to 2021 | Participants with ≥ 1 courses of antibiotics for ARIs of any cause |
| Pham, Hai | 2022 | ACTRN12613000743763 | 2014 to 2015 | Participants with antibiotic prescription ≥ 1 episodes |
| Rafiq, R. | 2022 | NCT02122627 | 2015 to 2019 | Participants with ≥ 1 episodes of antibiotic use |
| Tran, Bich | 2014 | ACTRN12609001063202 | 2010 to 2011 | Participants with antibiotic use |
| Wu, Zhenqiang | 2021 | ACTRN12611000402943 | 2011 to 2012 | The Proportion of participants prescribed ≥ 1 antibiotic |
| Avenell, A. | 2007 | ISRCTN51647438 | 1999 to 2002 | Participants with antibiotic use |

ARIs, acute respiratory infections; Episode, prescriptions dispensed within 21 days of another prescription comprised a single episode.
