# Supplementary figures and images for: The effect of vitamin D supplementation on antibiotic use: a meta-analysis based on randomized controlled trials
Source: Front Nutr. 2024 Nov 12;11:1502835. doi: 10.3389/fnut.2024.1502835 (PMC11588496; doi:10.3389/fnut.2024.1502835)

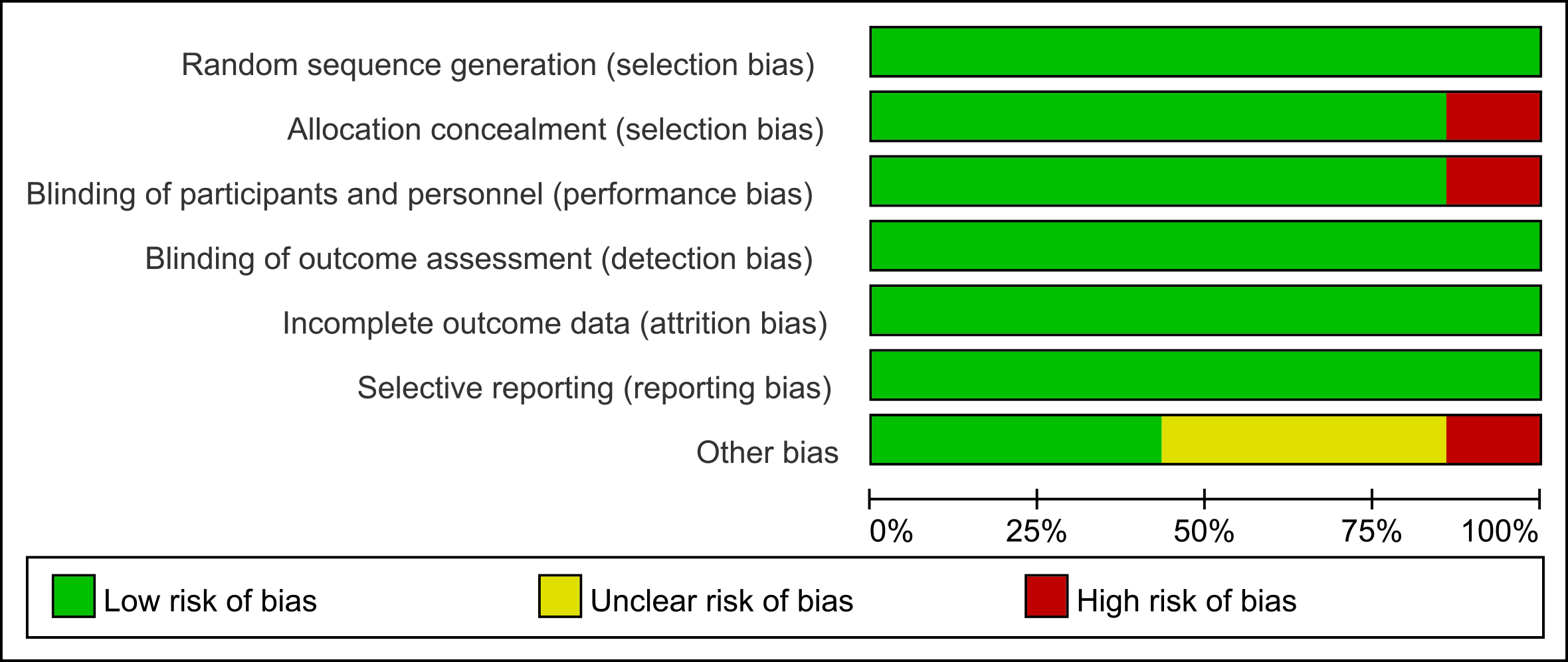

Supplement: SUPPLEMENTARY FIGURE S1 — Risk of bias graph for quality assessment of the included RCTs. [file Image_1.TIF]

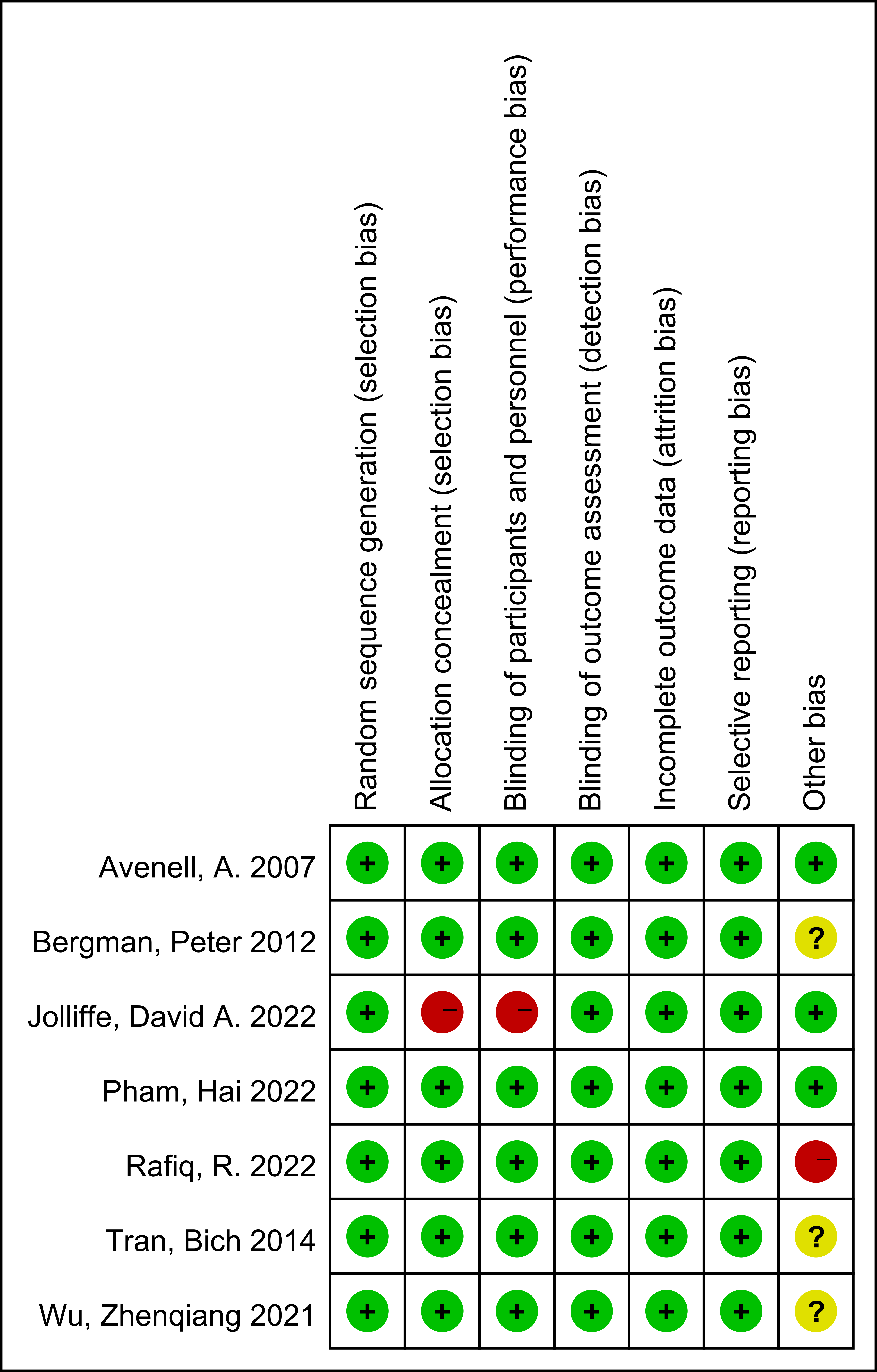

Supplement: SUPPLEMENTARY FIGURE S2 — Risk of bias summary for quality assessment of the included RCTs. [file Image_2.TIF]

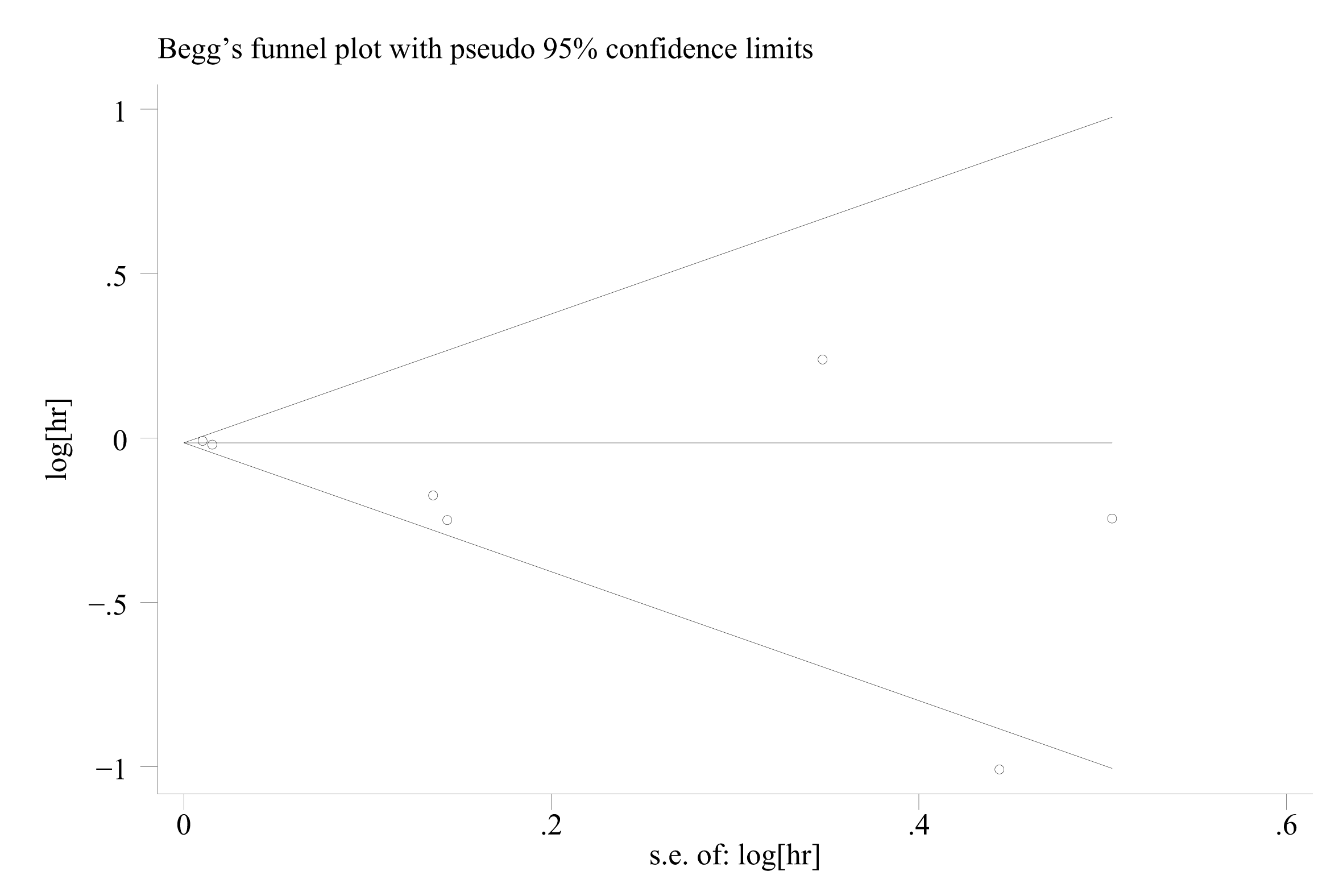

Supplement: SUPPLEMENTARY FIGURE S3 — Begg’s test for assessing publication bias of included RCTs (p=0.230). [file Image_3.TIF]

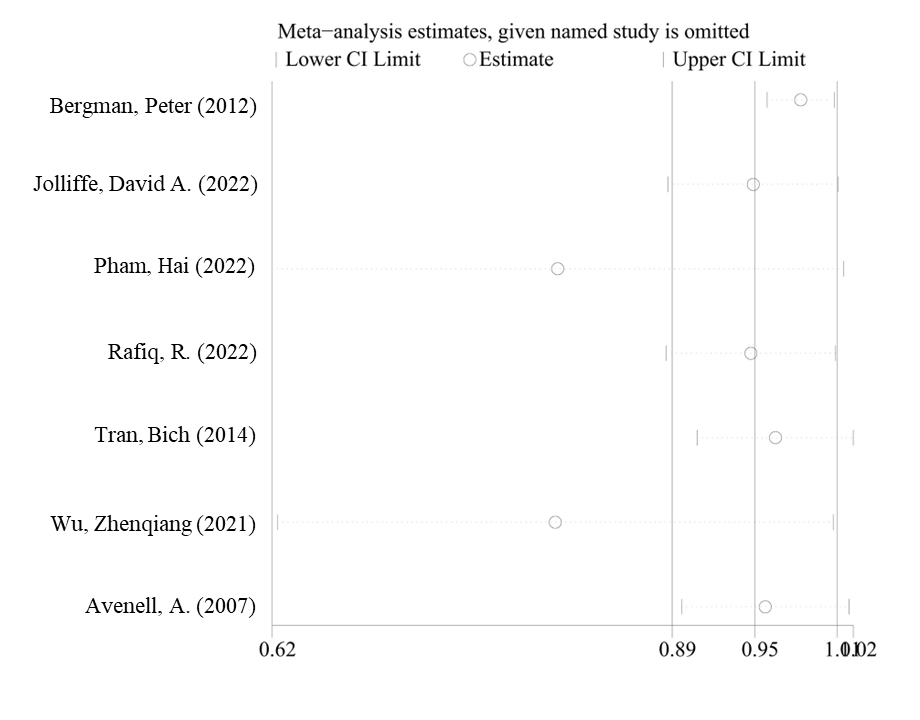

Supplement: SUPPLEMENTARY FIGURE S4 — Sensitivity analysis for testing the stability of statistical results. [file Image_4.TIF]
